# A Guided Mode Resonance Aptasensor for Thrombin Detection

**DOI:** 10.3390/s110908953

**Published:** 2011-09-19

**Authors:** Sheng-Fu Lin, Ting-Jou Ding, Jen-Tsai Liu, Chien-Chieh Lee, Tsung-Hsun Yang, Wen-Yih Chen, Jenq-Yang Chang

**Affiliations:** 1 Department of Optics and Photonics, National Central University, Jhongli 32001, Taiwan; E-Mails: 982406004@cc.ncu.edu.tw (S.-F.L.); 9424001@dop.ncu.edu.tw (T.-J.D.); thyang@ios.ncu.edu.tw (T.-H.Y.); 2 Department of Chemical and Materials Engineering, National Central University, Jhongli 32001, Taiwan; E-Mail: zhenchia.liu@gmail.com (J.-T.L.); 3 Optical Sciences Center, National Central University, Jhongli 32001, Taiwan; E-Mail: jjlee@cc.ncu.edu.tw (C.-C.L.); 4 Center for Dynamical Biomarkers and Translational Medicine, National Central University, Jhongli 32001, Taiwan

**Keywords:** aptamer, optical biosensor, guided mode resonance, thrombin, affinity constant

## Abstract

Recent developments in aptamers have led to their widespread use in analytical and diagnostic applications, particularly for biosensing. Previous studies have combined aptamers as ligands with various sensors for numerous applications. However, merging the aptamer developments with guided mode resonance (GMR) devices has not been attempted. This study reports an aptasensor based home built GMR device. The 29-mer thrombin aptamer was immobilized on the surface of a GMR device as a recognizing ligand for thrombin detection. The sensitivity reported in this first trial study is 0.04 nm/μM for thrombin detection in the concentration range from 0.25 to 1 μM and the limit of detection (LOD) is 0.19 μM. Furthermore, the binding affinity constant (K_a_) measured is in the range of 10^6^ M^−1^. The investigation has demonstrated that such a GMR aptasensor has the required sensitivity for the real time, label-free, *in situ* detection of thrombin and provides kinetic information related to the binding.

## 1. Introduction

In recent decades, biomarker detection, especially non-labeling assaying has shown great promise in diagnostic health care and the drug discovery process [1,2], particularly with regard to preventive measures aimed at cardiovascular diseases such as thrombotic disorders [3]. Previous studies have shown a strong correlation between these diseases and various biomarkers [4–6]. Human thrombin, a highly specific serine protease, is a biomarker that plays an important role in the coagulation cascade. Thrombin is involved in various pathogeneses, such as blood coagulation, incrustation, inflammation, and pulmonary metastasis [7,8]. Researchers have developed various kinds of biosensors for thrombin detection, including electrochemical [9,10], colorimetric [11–13], fluorescence [14], mass sensing transduction [15–17] and optical transduction sensors [18,19]. The optical method has attracted a great deal of attention because it provides a real-time, label-free, high-sensitivity, nondestructive mode of operation, and has the versatility required for devices in clinical applications [20]. Guided mode resonance (GMR) optical devices have been applied to biosensing since they were discovered to be sensitive to refractive index changes, and the first GMR biosensor design and experiments were reported in the early 2000s [21–23]. Recent studies have demonstrated that a newly recognized molecular species, the aptamer, has many advantages with regard to biosensing. This study develops a new thrombin biosensor using a home built optical GMR device, with the aptamer as the recognition species. The proposed GMR aptasensor is a label-free sensor capable of *in situ* real-time detection of binding kinetics.

The aptamer is a single strand DNA or RNA with specific binding capabilities with its ligand molecules. Aptamers were basically screen derived in the 1990s from the systematic evolution of ligands by the exponential enrichment during the so-called SELEX process [24–26]. Aptamers are able to bind to target molecules including amino acids, proteins, drugs, organic or inorganic molecules, or optical isomers with specificity and high affinity [27,28]. As a type of oligonucleotide, aptamers are easy to synthesize and modify on a large scale with functional groups or nanoparticles [7,13,29,30]. Aptamers have many applications, including separation, therapeutics, clinical diagnostics, and particularly in biosensing.

A GMR device is an optical filter that utilizes a grating on the top of a planar waveguide to reflect a specific wavelength of transmitted light [31]. This device produces a narrow resonance dip (a few nanometers wide) in the transmission spectrum. GMR sensors detect biomolecules and interactions between biomolecules by evanescent waves on the chip surface. The filtered wavelength shifts when the optical path length changes in the region of the evanescent waves. A GMR sensor offers the advantages of high-sensitivity [32], label-free, real-time detection, and high throughput [33].

Two aptamers have been developed for targeting thrombin in different sites with high affinity and specificity. These aptamers are 15 and 29 base oligonucleotides. The binding equilibrium constants (K_d_) were reported to be 26 nM and 0.5 nM, respectively [34,35]. This binding is similar to the binding of antibodies to their antigens. When thrombin binds to its aptamer ligand, the thrombin aptamer (TBA) plays the role of inhibitor, restricting the activity of the thrombin [36]. Studying the interaction between thrombin and its aptamer has great potential for clinical applications.

Recently, optimization for high sensitivity GMR sensor for various applications has been studied [37,38] and many reports shows that GMR devices are suitable for biosensors. On the other hand, aptamers show good storage, stability and flexibility for modification properties. Better detection sensitivity and specificity can be achieved by optimizing the nano-structure design of the GMR chip. This work aims on the combination of an GMR device and an aptamer for developing a new aptasensor. The investigation includes GMR surface modification, 29-mer TBA immobilization and kinetics studies between 29-mer TBA with thrombin. The proposed device achieves real-time and label-free detection of thrombin. The results are discussed in relation to detection sensitivity and the kinetic behavior of the thrombin binding with the aptamer.

## 2. Materials and Methods

### 2.1. GMR Fabrication

**Figure 1.**
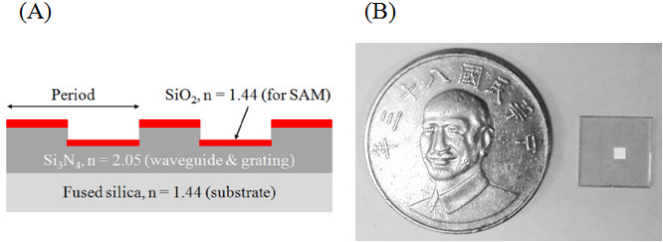
(**A**) Schematic representation of the GMR sensor structure with a 620 μm substrate of fused silica; both the waveguide and the grating were Si_3_N_4_. The single SiO_2_ layer on top of the grating was for surface modification. (**B**) The fabricated chip referencing with a coin. The bright spot is the structured sensing area which size is 1.5 × 1.5 mm^2^.

Figure 1 depicts the geometry of the GMR sensor, which has a 620 μm thick fused silica substrate, a waveguide and a grating layer, both made of Si_3_N_4_. A SiO_2_ layer for surface modification was deposited on top of the grating by plasma-enhanced chemical vapor deposition (PECVD, Unaxis/Nextral D200). The fabrication process began with a standard cleaning of the substrate using (1) acetone and (2) isopropyl alcohol, followed by (3) rinsing with deionized (DI) water. Each step took place in an ultrasonic cleaner for 3 min, followed by drying under a stream of pure nitrogen. After cleaning, a 180 nm layer of Si_3_N_4_ film was deposited by PECVD. Once the deposition was completed, a positive photoresist layer was spun onto the Si_3_N_4_. A one-dimensional grating for which the period is 950 nm and the filling factor is 0.5, was layered with an e-beam writer (Raith 150, Japan). After lithography, the photoresist was developed. The chip was etched using a high-density plasma etcher (HDP, Unaxis/Nextral 860L), and finally the residual photoresist was removed using standard cleaning procedures. The chip size is shown in Figure 1(B). The chip is 1 × 1 cm^2^ and the structured area is located at the center with size of 1.5 × 1.5 mm^2^. The detection area (structure area) is totally covered with the fluidic channel.

### 2.2. Reagents

The oligonucleotides were synthesized by MDBio Inc., Taiwan. The TBAs were modified with NH_2_ at the 5′ end and purified with HPLC. The sequence was 5′NH_2_-AGT CCG TGG TAG GGC AGG TTG GGG TGA CT-3′ [35]. Thrombin from human plasma was purchased from Sigma-Aldrich (St. Louis, MO, USA), along with 3-aminopropyltriethoxysilane (APTES), tris-HCl buffer (pH 7.4), and glutaraldehyde (GA). Salt for the buffer solutions, sodium phosphate monobasic, and sodium phosphate dibasic were obtained from Fluka. All reagents were standard grade.

### 2.3. Aptamer Immobilization

The aptamer was immobilized on the GMR surface based on a self-assembly monolayer method. First, 50 nm of SiO_2_ was deposited on top of the GMR chip using PECVD. The chip was cleaned with DI water and dried in a stream of N_2_. The chip was exposed to UV-ozone for 20 min to generate OH^−^ functional groups for self-assembly. It was subsequently soaked in a 3 mM APTES solution for 6 h to form a self-assembled monolayer (SAM). To avoid the formation of APTES clusters, the beaker was sealed with parafilm (Sigma-Aldrich). After 6 h, the chip was rinsed with ethanol, dipped into a 2% GA solution for 1 h, and then rinsed with DI water. After this the chip was immersed in a 1 μM TBA solution (diluted with phosphate buffered saline, PBS) overnight. Finally, the chip was stored in pure PBS at 4 °C.

### 2.4. Surface Characteristic

Wettability measurements were performed using a homemade contact angle meter. To measure the water contact angle (WCA), a 5 μL DI water drop was dripped on top of the chip. Images of the drops were captured by a CCD camera. For comparison, the GMR chip was replaced with fused silica slabs, but treated with the same modification process as for the GMR chip. The WCA measurements were performed after SiO_2_ deposition, after APTES modification, and after immobilizing the aptamer. Each WCA measurement was repeated three times.

Electron spectroscopy for chemical analysis (ESCA) was used to analyze the elements by detecting the binding energy between atoms. To identify the immobilization of the aptamers, the ESCA experiment focused on the phosphorus atoms. Specifically, they focused on the 2p orbit of phosphorus atoms with the three types of chips: bare fused silica, with SAM, and with immobilized aptamers. The sweeping range was from 140 to 120 eV with a step of 0.1 eV.

### 2.5. GMR Detection System

The GMR chip was mounted in a fluidic cell and illuminated with a broadband light source (Koheras superK, NTK Photonics, Denmark). The light source was connected to a collimator fiber, which collimated the incident light. A polarizer was placed immediately behind the collimated light source to convert the light into the transverse electric (TE) mode. Another collimator fiber was placed on the end to receive the transmitted light into an optical spectrum analyzer (OSA, MS9710B, Anritsu, Japan). The OSA was controlled by a computer in real-time using a general purpose interface bus (GPIB). The computer recorded any shifts in the wavelength of the filtered light.

### 2.6. Thrombin Assay

The buffer solution was 50 mM tris-HCl pH 7.4. The procedure comprised equilibrium baseline measurement, binding association, binding disassociation, surface regeneration, and re-baseline measurement in a temperature-controlled environment. Thrombin was dissolved in tris-HCl buffer at concentrations of 0.25 to 1.5 μM. The thrombin, pure tris-HCl buffer, and 2 M NaCl solutions were stored at 35 °C for 1 h before the experiment. The dimensions of the fluidic cell were 10 mm × 5 mm × 0.1 mm. A peristaltic pump moved the solution into the fluidic cell at a constant rate of 50 μL/min. However, due to limitations of the detection system setup, the tris-HCl buffer was pumped into the fluidic cell for 5 min for baseline measurement. The binding association rate constant was determined by pumping the thrombin/tris-HCl solution into the fluidic cell with the GMR chip modified by the TBA. Once the shifts of wavelength had stabilized, the tris-HCl buffer was again pumped for the disassociation process to removing the non-specifically bound thrombin, following by a regeneration step with the 2 M NaCl solution. These steps above were repeated with different concentrations of thrombin.

### 2.7. *In Situ* Binding Kinetic Measurements

This study adopted a simple Langmuir one-to-one adsorption isotherm for the kinetic analysis. The interactions of thrombin and TBA (A and B) are formulated as in Equation (1), and follow the rate equation given by Equation (2):

(1) $$A+B\overset{k_{a}}{\underset{k_{d}}{↔}}\mathrm{AB}$$

(2) $$d\left[\mathrm{AB}\right]/\mathrm{dt}=\left[A\right]\left[B\right]k_{a}-\left[\mathrm{AB}\right]k_{d}$$

where the terms *k_a_* and *k_d_* represent the binding association and dissociation rate constants. The signal from the biosensor (R) is proportional to the concentration of complex (AB), and the maximum signal (Rmax) is proportional to the initial concentration of (B) and is used for binding isotherm data. This means that Equation (2) can be rewritten as:

(3) $$\mathrm{dR}/\mathrm{dt}=k_{a}\left[A\right]R_{\text{max}}-\left(k_{a}\left[A\right]+k_{d}\right)R$$

Since the concentration of thrombin (A) vanishes due to the washing out of nonspecific binding in the dissociation phase. The rate constant *k_d_* can be determined from Equation (3), and the dissociation (washing) step sensorgram solely as:

(4) $$R=R_{0}e^{-tk_{d}}$$

Then, with the *k_d_* value obtained from Equation (4), the *k_a_* value can be derived or fitted for the sensorgram as:

(5) $$R=\left[A\right]k_{a}R_{\text{max}}\left[1-e^{-\left(\left(\left[A\right]k_{a}+k_{d}\right)t\right)}\right]/\left(\left[A\right]k_{a}+k_{d}\right)$$

The affinity constant (K_a_) is defined by *k_a_*/*k_d_* and is equal to the inverse of the dissociation equilibrium constant (K_d_) (Equation (6)), thus:

(6) $$K_{a}=k_{a}/k_{d}=1/K_{d}$$

## 3. Results and Discussion

### 3.1. GMR Device Property

**Figure 2.**
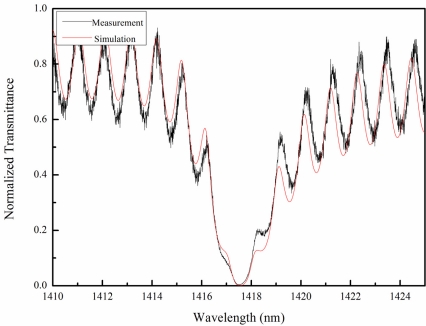
The transmission spectrum for the GMR chips (black) and the RCWA simulation (red) in an air environment. The resonance wavelength was 1,417.5 nm. The simulation parameters were in accordance with the AFM morphology results for the GMR chip. The side band ripples were the result of interference from the substrate.

The GMR chip morphology measurement results showed the period of the grating to be 957 nm and the depth of the grating to be approximately 55 nm. Ellipsometer measurement revealed the deposited Si_3_N_4_ to be 165 nm thick. The two layers of the waveguide and grating were 110 nm and 55 nm thick, respectively. The Gsolver software (Grating Solver Development Co., USA) was used to perform an optical simulation based on the measured geometry structure specifications. The algorithm of the Gsolver is based on the rigorous coupled-wave analysis (RCWA) method. Figure 2 shows the transmission spectrum, in air, as measured by OSA. Figure 2 also presents the simulated spectrum calculated by the Gsolver software. One dip of a specific wavelength in this spectrum indicates the GMR effect, or the resonance effect. The resonance wavelength was 1,417.5 nm, and the full width at half maximum (FWHM) was 2.5 nm in this case. It can be seen that both the simulation sample and the physical sample had ripple side bands. The period of the side band ripples was about 1 nm. This was due to interference between the two faces of the fused silica substrate, with a thickness of 620 μm and reflective index of 1.44. The OSA spectrum results agreed with the simulation results.

**Figure 3.**
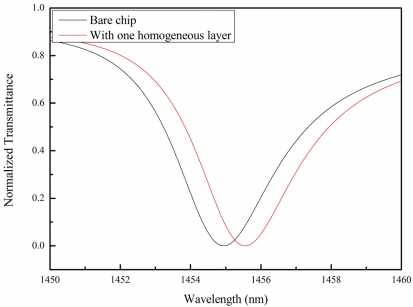
RCWA simulation results with (red) and without one homogeneous layer (black) on top of the GMR chip in water. The results show that the resonance wavelength shifted to a longer wavelength.

The effective refractive index on the chip surface changes when thrombin is bound with the immobilized aptamer. The shift in the effective refractive index is proportional to the amount of thrombin bound to the surface. The globular structure of the thrombin was observed to be approximately 8.8 nm in diameter [39]. Based on the geometry of the GMR chips and the thrombin mentioned above, the shift in the resonance wavelength in response in water was calculated. Simulations of the bare chip and one covered with a homogeneous 8.8 nm layer are shown in Figure 3. For a clearer presentation of the spectrum, in the simulation, the substrate of the GMR chip is removed to eliminate interference ripples. The resonance wavelength in the transmission spectrum shifted to a long wavelength (red shift). This was because the effective refractive index had changed to an optically denser medium. The optically denser medium provided a greater phase shift on the GMR sensor surface. Therefore, the transmission spectrum showed a red shift.

### 3.2. Surface Modification on the GMR Sensors

Each step of the surface modifications and aptamer immobilization showed different exposed functional groups on the surface of the chip. The WCA was measured to evaluate the wettability between each step of surface modifications. These measurements qualitatively indicated the progress and success of the surface modification. Table 1 shows that the unmodified bare fused silica surface exhibited high hydrophilicity, with a WCA of 20.5°. After SAM modification of the silica surface, as described in Section 2.3, the WCA increased to 51.7°. This large variation indicated that the NH_2_ group of SAM had made the surface more hydrophobic. These results agreed with previous reports on the silanization of glass slides [40]. The WCA decreased to 28.1° when the aptamer was successfully immobilized on SAM.

**Figure 4.**
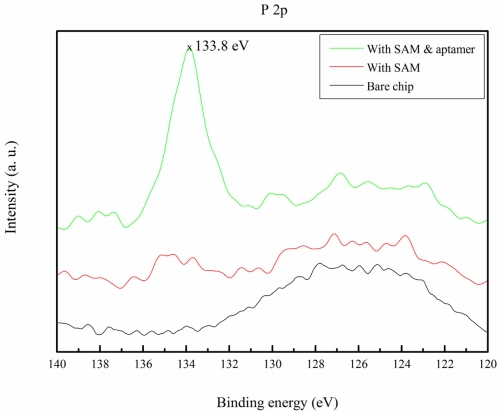
Bonding energy of bare fused silica (black), SAM (red), and chip with immobilized aptamer (green). The peak of the aptamer was at 133.8 eV, which is 6 times higher than that of other samples.

The immobilization of the aptamer was also verified by ESCA. The aptamer was an oligonucleotide of DNA, and the bonding energy of the DNA backbone phosphorus atom at 2p orbits (P 2p) was approximately 133.7 eV [41]. Neither the bare substrate, nor the SAM molecules have P 2p peaks. The ESCA results are shown in Figure 4 and the peak at 133.8 eV demonstrates that the aptamer was covalently immobilized.

### 3.3. GMR Real Time Thrombin Detection

**Figure 5.**
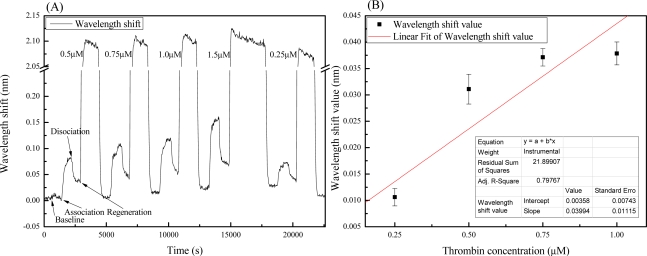
(**A**) Real-time sensorgram. The resonance wavelength shifted with different concentrations of thrombin. There were four steps in the detection process: first, pure tris-HCl buffer solution (baseline); second, buffer solution with thrombin (association); third, pure buffer again (dissociation); fourth, 2 M NaCl solution (regeneration). The four steps were repeated with different thrombin concentrations (0.25, 0.5, 0.75, 1, and 1.5 μM). (**B**) The resonance wavelength responses (shifts) of the GMR sensor to different concentrations of thrombin (form 0.25 to 1 μM). The error bars stand for the measurement noise which calculated from each baseline. The value of R^2^ is 0.8 and the sensitivity is calculated to be 0.04 nm/μM.

Figure 5(A) shows a sensorgram of the shift in adsorption wavelength with different thrombin concentrations. Each of the detection measurement processes includes the following steps: baseline (equilibrium), association (binding), disassociation, and regeneration. The thrombin concentrations used in this investigation were 0.25, 0.5, 0.75, 1, and 1.5 μM. Figure 5(B) shows the linear fitting between the thrombin concentrations (0.25–1 μM) and the binding responses of the wavelength shifts. The binding signal is defined as the wavelength dip value after dissociation, minus the baseline value. In the concentration range between 0.25–1 μM, the data can be fitted to a linear relation and shows an r-square value (R^2^) of 0.8. Results indicate that the sensitivity for thrombin detection in this range was 0.04 nm/μM. The standard deviation (σ) was calculated to be 0.0025 nm from the baselines. A 3 times value of σ stands for the noise signal which indicated the LOD of this system is 0.19 μM. Distinguishing the smallest thrombin concentration in the experiment (0.25 μM) would be adequate. Furthermore, Figure 5(B) provides an evidence of adsorption isotherms in the GMR system. This work aims to demonstrate the feasibility of the GMR as a biosensor and the reported sensitivity of this non-optimized chip is an order lower than that of other reported GMR sensord [32,33,37,38]. However, Abdulhalim has addressed some guidelines for enhancing the sensitivity of GMR based sensors [32]. The sensitivity improvement of the GMR aptasensor achieved through optimization is considerable.

The regeneration time in the experiment was 24 min, except for the regeneration of 1.5 μM thrombin. After the 24 min regeneration process, the signal wavelength values had decreased by 0.029 nm indicating that a number of thrombin-aptamer complexes may have been denatured by the 2 M NaCl solutions. In the regeneration process of 1.5 μM, the regeneration time was doubled and the signal wavelength value decreased by 0.039 nm. This showed that more thrombin-aptamer complexes had been denatured. This is because the shifts in wavelength were proportional to the amount of thrombin absorbed on the GMR chip surface. These results showed that the number of regenerated thrombin complexes was proportional to regeneration time.

### 3.4. GMR Aptasensor Determines the Kinetic Constants between Thrombin and TBA

This study also determined the binding kinetic constants by analyzing the real-time sensorgram with the Langmuir adsorption assumption. The sensorgram used purely represents the binding/adsorption concentration of the analytes. The Langmuir isotherm model was adapted is basically for its one-to-one binding simplicity. Also, discussion based on the obtained *k_a_* and *k_d_* for possible analysis of specific and non-specific binding is possible. As discussed above, the wavelength shifts are attributable to increased materials on the chip surface. In this study, wavelength shifts represent the formation of the aptamer-thrombin complex. Table 2 lists the constants. The association rate constants (*k_a_*) where shown to be on the order of 10^5^ M^−1^s^−1^, the dissociation rate constants (*k_d_*) were on the order of 10^−1^s^−1^, and the affinity constants (K_a_) were on the order of 10^6^ M^−1^. On the other hand, the K_a_ of the thrombin concentrations from 0.25 to 1 μM as calculated by the Langmuir linear regression is also gives a value on the order of 10^6^ M^−1^ which is the same order of fitting as from the real time sensorgram. The individual values of *k_a_* and *k_d_* may be affected by the diffusion, but the K_a_ value is a thermodynamic data result, no diffusion effect is involved. However, high values of the disassociation constant, *k_d_*, indicating high nonspecific binding between the immobilized aptamer and the surface with thrombin, are clearly present. This is why the K_a_ value in this report is lower than the value documented for other non-labeling sensors. The affinity constant for thrombin and its 29-mer TBA ranged from 10^7^ to 10^9^ according to different detection methods, such as quartz crystal microbalance (QCM), SELEX and electrochemical impedance spectroscopy (EIS) [15,35,42]. Based on these results and the different detection methods used, with more defined surface chemistry and the improvements of reducing non-specific binding by the incorporation of anti-fouling materials, such as PEG [43] or SBMA [44], the preliminary data presented by this first GMR measurement of aptasensor, suggest it is an applicable method for the kinetic analysis of biomolecular interactions.

## 4. Conclusions

This study demonstrated that a GMR aptasensor can be achieved by immobilization of aptamers on an optical GMR device. Measurements of thrombin with TBA were performed and the binding kinetics and binding constants were analysed. The GMR aptasensor has the capability of real-time, label free detection. The linear detection range for thrombin was 0.25–1 μM, and the LOD was 0.19 μM. Kinetic analysis demonstrated the affinity constant was in the 10^6^ M^−1^ range. However, there are some issues that need to be overcome, such as the diffusion effect, the non-specific binding and the sensitivity optimization. Through this first trial of an GMR aptasensor and other research results, the GMR aptasensor system shows a low-cost and potential for high throughput and easy application to different targets that makes the GMR aptasensor a good choice for diagnostic or analytical applications in the future.

## Figures and Tables

**Table 1.** Water contact angle measurement showing the different stages of surface modification from the bare substrate to SAM and immobilization of the aptamer.

**Surface modification**	**Fused silica**	**APTES modified**	**Aptamer modified**
WCA	20.7°	51.7°	28.1°

**Table 2.** TBA and thrombin interaction kinetic constants. The constants were calculated using the real-time sensorgram in Figure 5(A).

**Concentration (μM)**	***k_a_* (10^5^ M^−1^s^−1^)**	***k_d_* (10^−1^ s^−1^)**	**K_a_ (10^6^ M^−1^)**
0.25	2.06	1.05	1.95
0.50	1.52	0.61	2.50
0.75	1.83	0.30	6.18
1.00	0.18	1.54	0.12
1.50	0.20	1.33	0.15

## References

[1] Chen L, Bao CC, Yang H, Li D, Lei C, Wang T, Hu HY, He M, Zhou Y, Cui DX (2011). A prototype of giant magnetoimpedance-based biosensing system for targeted detection of gastric cancer cells. Biosens. Bioelectron.

[2] Zhou Y, Yang H, Chen L, Lei C, Zhang J, Li D, Zhou ZM, Bao CC, Hu HY, Chen XA (2010). Giant magnetoimpedance-based microchannel system for quick and parallel genotyping of human papilloma virus type 16/18. Appl Phys Lett.

[3] Gopinath SCB (2008). Anti-coagulant aptamers. Thromb. Res.

[4] Arntz Y, Seelig JD, Lang HP, Zhang J, Hunziker P, Ramseyer JP, Meyer E, Hegner M, Gerber C (2003). Label-free protein assay based on a nanomechanical cantilever array. Nanotechnology.

[5] Hasegawa K, Ono K, Yamada M, Naiki H (2002). Kinetic modeling and determination of reaction constants of Alzheimer’s beta-amyloid fibril extension and dissociation using surface plasmon resonance. Biochemistry.

[6] Petrou PS, Ricklin D, Zavali M, Raptis I, Kakabakos SE, Misiakos K, Lambris JD (2009). Real-time label-free detection of complement activation products in human serum by white light reflectance spectroscopy. Biosens. Bioelectron.

[7] Ding CF, Ge Y, Lin JM (2010). Aptamer based electrochemical assay for the determination of thrombin by using the amplification of the nanoparticles. Biosens. Bioelectron.

[8] Hernandez-Rodriguez NA, Correa E, Contreras-Paredes A (1997). Thrombin: A new useful factor in the early diagnosis of pulmonary metastasis. Rev. Inst. Nal. Cancerol.

[9] Cai H, Lee TMH, Hsing IM (2006). Label-free protein recognition using an aptamer-based impedance measurement assay. Sens. Actuat. B.

[10] Lee JA, Hwang S, Kwak J, Park SI, Lee SS, Lee KC (2008). An electrochemical impedance biosensor with aptamer-modified pyrolyzed carbon electrode for label-free protein detection. Sens. Actuat. B.

[11] Higuchi A, Siao YD, Yang ST, Hsieh PV, Fukushima H, Chang Y, Ruaan RC, Chen WY (2008). Preparation of a DNA aptamer-Pt complex and its use in the colorimetric sensing of thrombin and anti-thrombin antibodies. Anal. Chem.

[12] Wang YL, Li D, Ren W, Liu ZJ, Dong SJ, Wang EK (2008). Ultrasensitive colorimetric detection of protein by aptamer—Au nanoparticles conjugates based on a dot-blot assay. Chem Commun.

[13] Zhang ZX, Wang ZJ, Wang XL, Yang XR (2010). Magnetic nanoparticle-linked colorimetric aptasensor for the detection of thrombin. Sens. Actuat. B.

[14] Wang WJ, Chen CL, Qian MX, Zhao XS (2008). Aptamer biosensor for protein detection based on guanine-quenching. Sens. Actuat. B.

[15] Hianik T, Ostatna V, Zajacova Z, Stoikova E, Evtugyn G (2005). Detection of aptamer-protein interactions using QCM and electrochemical indicator methods. Bioorg. Med. Chem. Lett.

[16] Jung A, Gronewold TMA, Tewes M, Quandt E, Berlin P (2007). Biofunctional structural design of SAW sensor chip surfaces in a microfluidic sensor system. Sens. Actuat. B.

[17] Schlensog MD, Gronewold TMA, Tewes M, Famulok M, Quandt E (2004). A Love-wave biosensor using nucleic acids as ligands. Sens. Actuat. B.

[18] Liao W, Wei F, Liu D, Qian MX, Yuan G, Zhao XS (2006). FTIR-ATR detection of proteins and small molecules through DNA conjugation. Sens. Actuat. B.

[19] Zhu HY, Suter JD, White IM, Fan XD (2006). Aptamer based microsphere biosensor for thrombin detection. Sensors.

[20] Luppa PB, Sokoll LJ, Chan DW (2001). Immunosensors—principles and applications to clinical chemistry. Clin. Chim. Acta.

[21] Wawro D, Tibuleac S, Magnusson R, Liu H (2000). Optical fiber endface biosensor based on resonances in dielectric waveguide gratings. Proc. SPIE.

[22] Kikuta H, Maegawa N, Mizutani A, Iwata K, Toyota H (2001). Refractive index sensor with a guided-mode resonant grating filter. Proc. SPIE.

[23] Cunningham B, Li P, Lin B, Pepper J (2002). Colorimetric resonant reflection as a direct biochemical assay technique. Sens. Actuat. B.

[24] Ellington AD, Szostak JW (1990). *In vitro* selection of RNA molecules that bind specific ligands. Nature.

[25] Robertson DL, Joyce GF (1990). Selection *in vitro* of an RNA enzyme that specifically cleaves single-stranded-DNA. Nature.

[26] Tuerk C, Gold L (1990). Systematic evolution of ligands by exponential enrichment—RNA ligands to bacteriophage-T4 DNA-polymerase. Science.

[27] Jenison RD, Gill SC, Pardi A, Polisky B (1994). High-resolution molecular discrimination by RNA. Science.

[28] Tombelli S, Minunni A, Mascini A (2005). Analytical applications of aptamers. Biosens. Bioelectron.

[29] Maehashi K, Matsumoto K (2009). Label-free electrical detection using carbon nanotube-based biosensors. Sensors.

[30] Chiu TC, Huang CC (2009). Aptamer-functionalized nano-biosensors. Sensors.

[31] Moharam MG, Gaylord TK (1981). Rigorous coupled-wave analysis of planar-grating diffraction. J. Opt. Soc. Am.

[32] Abdulhalim I, Bock WJ, Gannot I, Tanev S (2008). Biosensing configurations using guided wave resonant structures. Optical Waveguide Sensing and Imaging.

[33] Choi CJ, Cunningham BT (2007). A 96-well microplate incorporating a replica molded microfluidic network integrated with photonic crystal biosensors for high throughput kinetic biomolecular interaction analysis. Lab Chip.

[34] Bock LC, Griffin LC, Latham JA, Vermaas EH, Toole JJ (1992). Selection of single-stranded-DNA molecules that bind and inhibit human thrombin. Nature.

[35] Tasset DM, Kubik MF, Steiner W (1997). Oligonucleotide inhibitors of human thrombin that bind distinct epitopes. J. Mol. Biol.

[36] Tsiang M, Jain AK, Dunn KE, Rojas ME, Leung LLK, Gibbs CS (1995). Functional mapping of the surface residues of human thrombin. J. Biol. Chem.

[37] Abdulhalim I (2009). Optimized guided mode resonant structure as thermooptic sensor and liquid crystal tunable filter. Chin. Opt. Lett.

[38] Krasnykov O, Auslender M, Abdulhalim I (2001). Optimizing the guided mode resonance structure for optical sensing in water. Phys. Express.

[39] Johnson DJD, Adams TE, Li W, Huntington JA (2005). Crystal structure of wild-type human thrombin in the Na^+^-free state. Biochem. J.

[40] Cras JJ, Rowe-Taitt CA, Nivens DA, Ligler FS (1999). Comparison of chemical cleaning methods of glass in preparation for silanization. Biosens. Bioelectron.

[41] Vilar MR, do Rego AMB, Ferraria AM, Jugnet Y, Nogues C, Peled D, Naaman R (2008). Interaction of self-assembled monolayers of DNA with electrons: HREELS and XPS studies. J. Phys. Chem. B.

[42] Li XX, Shen LH, Zhang DD, Qi HL, Gao Q, Ma F, Zhang CX (2008). Electrochemical impedance spectroscopy for study of aptamer-thrombin interfacial interactions. Biosens. Bioelectron.

[43] Pollet J, Delport F, Janssen KPF, Jans K, Maes G, Pfeiffer H, Wevers M, Lammertyn J (2009). Fiber optic SPR biosensing of DNA hybridization and DNA-protein interactions. Biosens. Bioelectron.

[44] Chang Y, Liao SC, Higuchi A, Ruaan RC, Chu C, Chen WY (2008). A highly stable nonbiofouling surface with well-packed grafted zwitterionic polysulfobetaine for plasma protein repulsion. Langmuir.

